# Hermeticity Analysis on SiC Cavity Structure for All-SiC Piezoresistive Pressure Sensor

**DOI:** 10.3390/s21020379

**Published:** 2021-01-07

**Authors:** Baohua Tian, Haiping Shang, Lihuan Zhao, Dahai Wang, Yang Liu, Weibing Wang

**Affiliations:** 1 Institute of Microelectronics of the Chinese Academy of Sciences, Beijing 100029, China; tianbaohua@ime.ac.cn (B.T.); zhaolihuan@ime.ac.cn (L.Z.); wangdahai@ime.ac.cn (D.W.); liuyang2@ime.ac.cn (Y.L.); wangweibing@ime.ac.cn (W.W.); 2 University of Chinese Academy of Sciences, Beijing 100049, China

**Keywords:** all-silicon carbide (SiC), sealed cavity structure, room temperature bonding, hermeticity, piezoresistive pressure sensor

## Abstract

The hermeticity performance of the cavity structure has an impact on the long-term stability of absolute pressure sensors for high temperature applications. In this paper, a bare silicon carbide (SiC) wafer was bonded to a patterned SiC substrate with shallow grooves based on a room temperature direct bonding process to achieve a sealed cavity structure. Then the hermeticity analysis on the SiC cavity structure was performed. The microstructure observation demonstrates that the SiC wafers are tightly bonded and the cavities remain intact. Moreover, the tensile testing indicates that the tensile strength of bonding interface is ~8.01 MPa. Moreover, the quantitative analysis on the airtightness of cavity structure through leakage detection shows a helium leak rate of ~1.3 × 10^−10^ Pa⋅m^3^/s, which satisfies the requirement of the specification in the MIL-STD-883H. The cavity structure can also avoid an undesirable deep etching process and the problem caused by the mismatch of thermal expansion coefficients, which can be potentially further developed into an all-SiC piezoresistive pressure sensor employable for high temperature applications.

## 1. Introduction

High temperature pressure sensors have wide application requirements in the petrochemical, aerospace, and automotive fields [1]. In the past few decades, silicon has become the dominant material for pressure sensors. However, due to the p–n junction failure and the loss of mechanical reliability in high temperature environments, silicon-based pressure sensors cannot meet the pressure measurements for high temperature applications [2,3,4]. Silicon carbide (SiC) is considered one of the most promising materials in high temperature pressure sensors due to its excellent characteristics, including wide band-gap, high breakdown electric field, high thermal stability, and high chemical inertness [5,6,7]. With the continuous breakthrough of SiC wafer fabrication and process technology, fruitful research on all-SiC piezoresistive pressure sensors has been carried out in recent years [8,9,10], which can avoid the thermal stress caused by the mismatch in the thermal expansion coefficients between different materials to improve the reliability and stability of sensor in high temperature environment [11].

**Figure 1:**
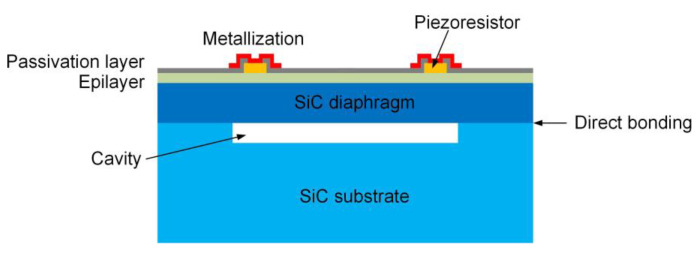
Structure schematic of the all-silicon carbide (SiC) piezoresistive pressure sensor.

The hermeticity performance of the cavity structure is highly significant for high temperature absolute pressure sensors, which not only achieve isolation from the external environment to prevent the contamination of the diaphragm, but also provide the stable reference pressure for external pressure to obtain the accurate sensor output [12,13,14]. In the high temperature environment, the thermal expansion of residual gas inside the cavity results in the pressure change and diaphragm deformation, which causes the temperature drift of sensor output and affects the measurement accuracy [15]. Furthermore, the voids and cracks of the cavity structure could cause stress concentration to reduce the reliability and operating life of the sensor. Therefore, the formation of a sealed cavity structure is one of the key technological issues for all-SiC piezoresistive pressure sensors [10]. The traditional methods have been developed in which a diaphragm fabricated by deep cavity machining on SiC substrate is bonded to another SiC wafer. For instance, the molten salt corrosion method to chemically etch SiC in molten potassium hydroxide (KOH) typically requires expensive Pt beakers and masks. Photoelectrochemical etching takes diluted hydrofluoric acid (HF) as the electrolyte and increases the hole generation through ultraviolet illumination. However, the need for electrical contacts during etching process restricts its application in SiC machining [13]. Moreover, ultrasonic machining, mechanical grinding and laser scribing can effectively shorten the etching time. However, obvious scratches and poor surface quality reduce the reliability of the structure in the high temperature environment [10,11,16]. Plasma etching is widely used in the formation of the cavity structure, which has the disadvantages of time-consuming of deep etching and poor accuracy of diaphragm thickness [5]. To solve the problems in the above processes, SiC diaphragm bonding onto a patterned SiC substrate with shallow grooves is another choice to form a sealed cavity structure, which could be used for the construction of an all-SiC piezoresistive pressure sensor, as shown in Figure 1. The SiC diaphragm with low surface roughness could be an SiC layer fabricated by wafer thinning and chemical mechanical polishing (CMP) with the support of a carrier wafer. Moreover, the depth of the cavity can be determined according to the target measurement range of the pressure sensor without deep etching. Most importantly, for the method of SiC bonding, Liang et al. [17] fabricated the bonded SiC structure with cavity based on direct pre-bonding in deionized water at room temperature, and then annealing at 1573 K for 3 h. Yushin et al. [18] achieved direct bonding of SiC wafers under a uniaxial stress of 20 MPa with annealing at 1073–1373 K for 15 h in ultrahigh vacuum. These methods may result in a huge energy consumption. In addition, wafer indirect bonding uses an intermediate layer including Ni and SiO_2_ [19,20]. However, the introduction of the intermediate layer may limit the application of SiC. In our experiment, the room temperature direct bonding technique avoids the energy consumption caused by a high temperature bonding process and the thermal stress caused by the mismatch in thermal expansion coefficients of different materials.

To realize the above piezoresistive pressure sensor, it is worth demonstrating the formation of SiC cavity structure. In this paper, a bare SiC wafer is bonded to another patterned SiC substrate with etched shallow grooves based on the room temperature direct bonding process to form a sealed cavity structure. The experiment could serve as a demonstration of the idea presented above. Further, the hermeticity performance of the cavity structure is respectively analyzed by means of microstructure evaluation, tensile strength testing and leakage detection.

## 2. Theory and Experiment

### 2.1. Theory

Due to the limitation of actual manufacturing process, the sealed cavity structure cannot achieve an absolute vacuum, which is mainly caused by gas leakage. The flow of gas through the leakage channel can be expressed as [21],

(1) $$F=\frac{L}{P_{A}-P}$$

where *F* is conductance of the leak channel, *L* is the true leak rate of air, *P* is the pressure inside the cavity, and *P_A_* is the atmospheric pressure. The pressure change per time inside the cavity can be estimated by

(2) $$\frac{dP}{dt}=\frac{FP}{V}$$

where *V* is the internal volume of the sealed cavity. Based on Formulas (1) and (2), we get

(3) $$\left(\ln P_{t}-\frac{P_{t}}{P_{A}}\right)-\left(\ln P_{0}-\frac{P_{0}}{P_{A}}\right)=\frac{Lt}{VP_{A}}$$

where *P*_0_ is the initial pressure inside the cavity and *P_t_* is the pressure inside the cavity at time *t*. The gas inside the cavity follows the state equation of ideal gas, which is expressed as

(4) $$P_{t}V=nRT_{1}$$

where *n* is amount of substance, *R* is gas constant, and *T*_1_ is initial ambient temperature.

In the high temperature and pressure environment, the variation in the cavity volume causes a change of pressure inside the cavity. The gas equilibrium equation can be expressed as

(5) $$P_{m}\left(V-\Delta V\right)=nRT_{2}$$

where Δ*V* is the change in cavity volume and *T*_2_ is ambient temperature. Based on the Formulas (4) and (5), we get

(6) $$P_{m}=P_{t}\frac{T_{2}}{T_{1}}\frac{V}{V-\Delta V}$$

**Figure 2:**
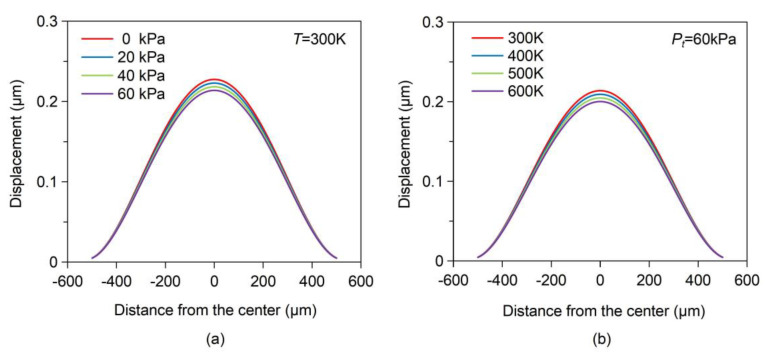
(**a**) Variation in diaphragm deformation with pressure inside the cavity. (**b**) Variation in diaphragm deformation with ambient temperature.

The variation in cavity volume can be calculated by the following formula:

(7) $$\Delta V=\int_{0}^{2\pi }d\theta \int_{0}^{a}w\left(r\right)rdr$$

where *w*(*r*) is the displacement of diaphragm. The deflection of a circular diaphragm can be expressed by

(8) $$w\left(r\right)=\frac{3\left(1-v^{2}\right){\left(a^{2}-r^{2}\right)}^{2}}{16Et^{3}}\left(P_{E}-P_{m}\right)$$

where *r* is the distance to the center of diaphragm, *a* and *t* are the radius and thickness of diaphragm, *v* and E are the Poisson’s ratio and Young’s modulus, and *P_E_* is the applied external pressure. For instance, for a circular diaphragm of 1000 μm in diameter and 50 μm in thickness, the effect of pressure inside the cavity, *P_t_*, and temperature, *T*, on the diaphragm deformation under a uniform pressure of 1MPa was analyzed, respectively, as shown in Figure 2. Moreover, the influence of thermal expansion of the SiC material caused by temperature variation on the diaphragm deformation is not considered. It is shown that the diaphragm displacement decreases with the increase of *P_t_* at room temperature, which indicates that the gas leakage of the cavity affects the measurement accuracy of the pressure sensor. Moreover, the thermal expansion of the gas inside the cavity causes the pressure to increase with the increasing temperature, resulting in the temperature drift of sensor output. Therefore, the hermeticity performance of the cavity structure is significantly important for the stability of the high temperature pressure sensor.

### 2.2. Experiment

**Figure 3:**
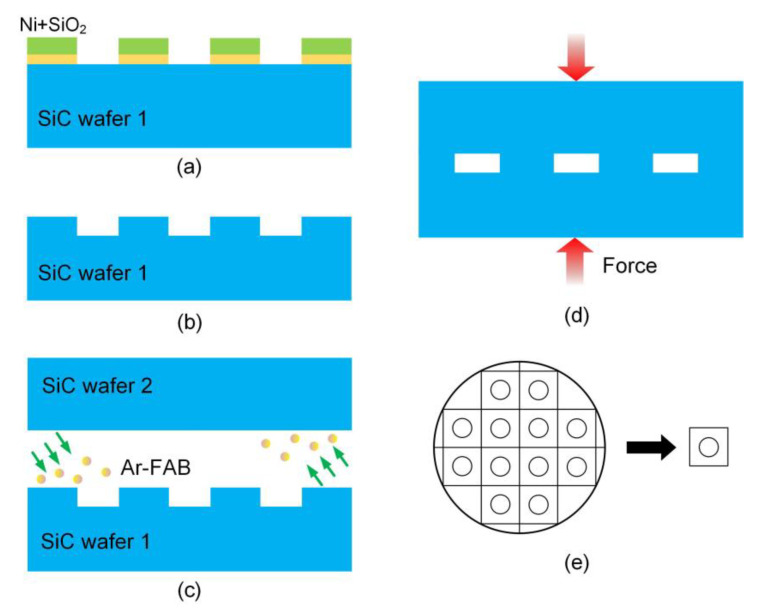
Fabrication process schematic of the SiC cavity structure: (**a**) etching of Ni and SiO_2_; (**b**) cavity etched by ICP; (**c**) Ar-FAB bombardment; (**d**) wafers contact each other under ~20MPa; (**e**) bonded wafers cut into pieces.

N-type 4H-SiC wafers (~350-μm-thick) were used for SiC cavity structure preparation in this experiment. Micropipes, one of the structural defects in SiC material, are basically hollow tube defects extending along the *c* axis. To avoid the influence of micropipes on the hermeticity of the cavity structure, the micropipe density of the SiC wafers was less than 1 cm^−2^. The fabrication process schematic of the SiC cavity structure is shown in Figure 3. First, a ~100 nm-thick SiO_2_ film and a ~500 nm-thick Ni mask were deposited on a SiC wafer respectively. The pattern of the etched areas was achieved by photolithography process, and the etched areas of SiC wafer were exposed after wet etching of Ni mask and SiO_2_ film, as shown in Figure 3a. After that, the etching of SiC was carried out in an inductively coupled plasma (ICP) etching system using SF_6_ and O_2_ gas. The etching rate was 360 nm per minute and shallow circular grooves with a depth of 20 μm were obtained after the wet etching of Ni and SiO_2_, as shown in Figure 3b. The SiO_2_ film forms an isolation layer to prevent the formation of contamination between Ni mask and the oxide film on SiC wafer surface. Moreover, the contamination and impurities left on SiC surface after the etching process seriously increase the surface roughness and affect the hermeticity performance of the cavity structure. Our team developed a method to provide excellent SiC surface preparation for a subsequent bonding process through ultrasonic cleaning and oxygen plasma bombardment [22]. The root-mean-square (RMS) surface roughness was confirmed as ~0.0962 nm by atomic force microscopy (AFM).

Surface activated bonding (SAB) was employed to bond the patterned SiC wafer to another bare wafer at room temperature. First, the SiC wafers were fixed on electrostatic chucks. Argon fast atom beam (Ar-FAB) was performed to remove contamination and the oxide layer from the bonding surfaces in an ultrahigh-vacuum environment, as shown in Figure 3c. The treated surfaces became so active that they could spontaneously form chemical bonds with each other at room temperature. Then the activated surfaces were brought into contact with each other under ~20MPa, as shown in Figure 3d. Room temperature direct bonding technology avoids the problem caused by the mismatch in thermal expansion coefficients and improves the stability of the sensor in high temperature environments [23]. Finally, the bonded wafers were diced into 15 mm × 15 mm samples for subsequent tests, as shown in Figure 3e.

Subsequently, the hermeticity performance of SiC cavity structure was analyzed. The quality of the bonding interface and the cavity structure were characterized by microstructure evaluation. Moreover, the tensile testing was performed to determine the bonding strength of the cavity structure, which can be calculated using the following formula:

(9) $$\sigma_{\max}=\frac{F_{\max}}{S}$$

where *S* is the area of the bonding interface and *F*_max_ is the maximum tension when the bonding interface is pulled off. Further, the leak rate is an intuitive parameter to reflect the hermeticity performance of the cavity structure. According to the specifications prescribed by the MIL-STD-883H method 1014.13 standard [24], a fine leak detection utilizing helium mass spectrometry and a coarse leak detection utilizing fluorine oil were carried out to analyze the tightness of cavity structure. The equivalent standard leak rate of the cavity structure can be calculated by the Howell–Mann equation

(10) $$R_{1}=\frac{LP_{He}}{P_{A}}\sqrt{\frac{M_{A}}{M_{He}}}\left[1-\exp\left(\frac{Lt_{E}}{VP_{A}}\sqrt{\frac{M_{A}}{M_{He}}}\right)\right]\exp\left(-\frac{Lt_{R}}{VP_{A}}\sqrt{\frac{M_{A}}{M_{He}}}\right)$$

where *R*_1_ is the measured leak rate of helium, *L* is the equivalent standard leak rate of air, *P_He_* is the pressurized pressure of helium, *M_A_* is the molecular weight of air, *M_He_* is the molecular weight of helium gas, *t_E_* is the time of expose to *P_He_*, *t_R_* is the dwell time between unloading from the helium chamber and leak detection.

## 3. Results and Discussion

### 3.1. Microstructure Evaluation

**Figure 4:**
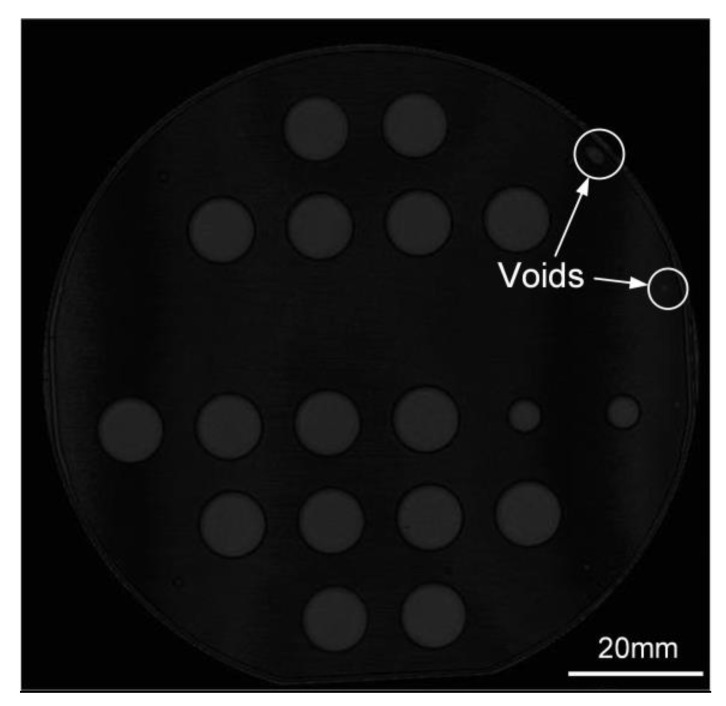
Scanning acoustic microscopy (SAM) image of the bonded SiC wafer.

The contamination and impurities adsorbed on the SiC wafer surface can form gaps and voids at the bonding interface, which directly affects the hermeticity performance of cavity structure. Therefore, the bonding uniformity of the bonded SiC wafer was examined through scanning acoustic microscopy (SAM) before cutting the wafers, as shown in Figure 4. The black areas indicate tight bonding and the gray ones represent sealed cavities and some voids. Nearly the whole wafer is bonded very well, except for a few voids around the wafer edges. The voids mainly result from particle contamination on the wafer surfaces, which might not be removed completely during the cleaning process. Moreover, the wafer surface was observed through optical microscopy, which indicates that the influence of micropipes on the hermeticity performance of cavity structure can be eliminated because there is no micropipe in critical areas, especially cavities.

**Figure 5:**
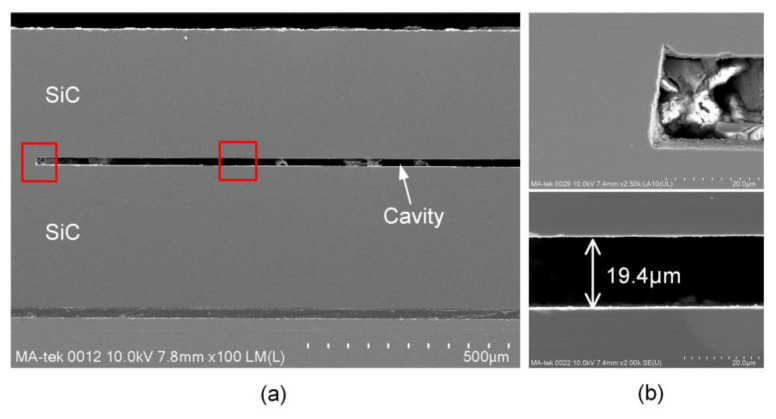
SEM images of the SiC cavity structure: (**a**) cross-sectional image of the bonded structure; (**b**) the magnified rectangular areas in (**a**).

Excellent interface characteristics are important for the hermeticity performance of cavity structure. The voids at the bonding interface are likely to become a channel connecting the cavity to the external environment and the cracks can cause stress concentration to reduce the reliability of the cavity structure in the high temperature environment. To observe the internal morphology, the bonded SiC sample was cut along the cross-section in this experiment. The bonding interface and cavity structure were observed through scanning electron microscope (SEM), as shown in Figure 5. It is indicated that there is no clear void or crack in the homogeneous and seamless interface. Moreover, the cavity structure is intact and still maintains at a height of about ~19.4 μm, which can meet the requirement of cavity structure for pressure sensor applications. The debris inside the cavity is left during the wafer dicing.

**Figure 6:**
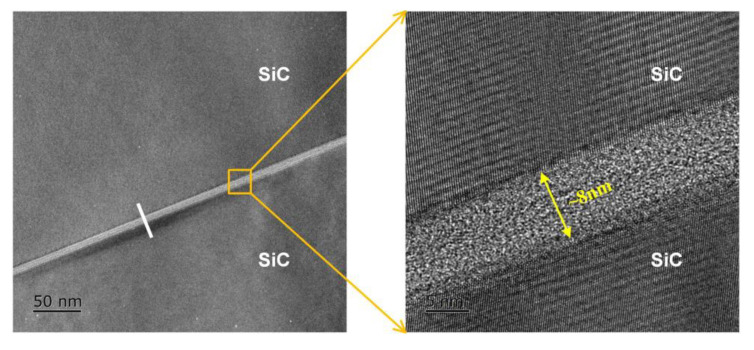
TEM image of the SiC–SiC bonding interface.

**Figure 7:**
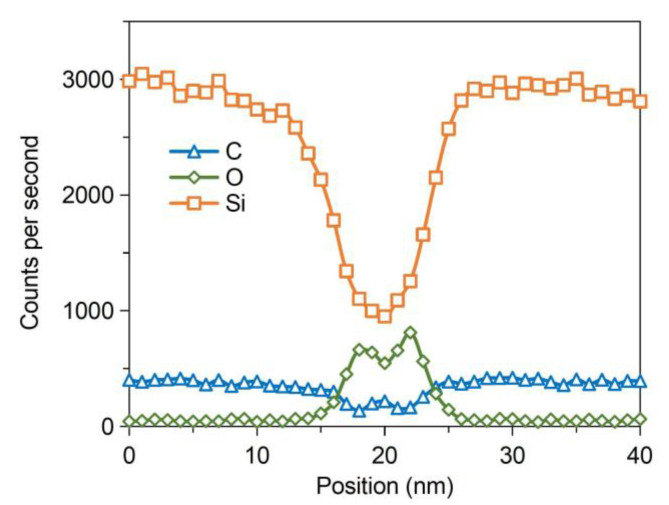
Energy-dispersive X-ray spectroscopy (EDX) line scanning analysis of the SiC–SiC bonding interface.

The interface characteristics were further analyzed by transmission electron microscopy (TEM), as displayed in Figure 6. There is an amorphous intermediate layer with a thickness of ~8 nm at the bonding interface between the bonded SiC structure, which has no lattice fringe and differs from the adjacent SiC material. Therefore, it can be inferred that the amorphous layer is formed on the SiC surfaces after Ar-FAB bombardment. After that, the element analysis along the white line in Figure 6 was performed by energy-dispersive X-ray spectroscopy (EDX), as shown in Figure 7. The counts per second on the y-axis represents the detected amount of atoms. It is indicated that both the Si spectra and O spectra in the intermediate layer have significant changes. Compared with the adjacent SiC material, it is found that the amount of Si is significantly decreased and the amount of C is slightly decreased, which is due to that the preferential sputtering occurs in SiC during Ar-FAB bombardment and the sputtering yield of Si is higher than that of C. Moreover, a peak in the O spectra indicates that the oxide layers on SiC surfaces are not completely removed by Ar-FAB bombardment, which might be unfavorable to the quality of bonding interface.

### 3.2. Tensile Strength Testing

**Figure 8:**
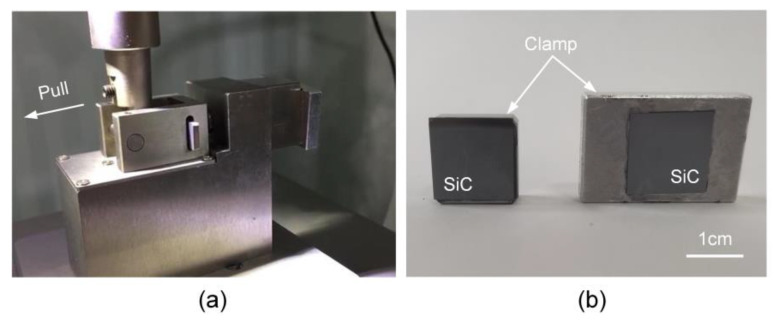
Bonding strength testing of SiC cavity structure: (**a**) tensile testing machine; (**b**) fracture of the bonding structure.

The strength of bonding interface is one of the key parameters to evaluate the sealed cavity structure, which has an impact on the stability and reliability of pressure sensors under the extreme environment. Weaker interface could normally not survive during processing and operating. In this experiment, tensile testing was performed to measure the bonding strength of the cavity structure. The sample was attached to the clamps with epoxy resin adhesive and loaded into the tensile testing machine (TRY-Precision MFM1500), as shown in Figure 8a. The tensile speed was 300 μm/s and the applied tensile force was recorded when the bonding interface was pulled off. Figure 8b illustrates the appearance of the sample after separation. It can be seen that the surfaces after separation were smooth, indicating that the fracture basically happened at the bonding interface. According to Formula (9), the average tensile strength of the cavity structure is ~8.01 MPa. The value is above the minimum acceptable bonding strength of 4–5 MPa required for most Micro-Electro-Mechanical Systems (MEMS) devices [25], which is sufficient for the bonding of diaphragm and back cavity in the pressure sensor.

### 3.3. Leakage Detection

**Figure 9:**
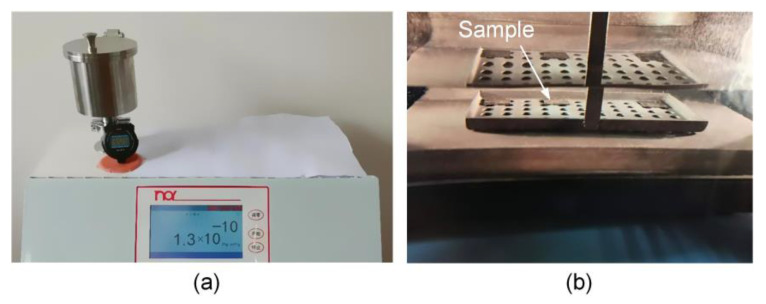
Leakage detection of SiC cavity structure: (**a**) the fine leak detection; (**b**) the coarse leak detection.

**Figure 10:**
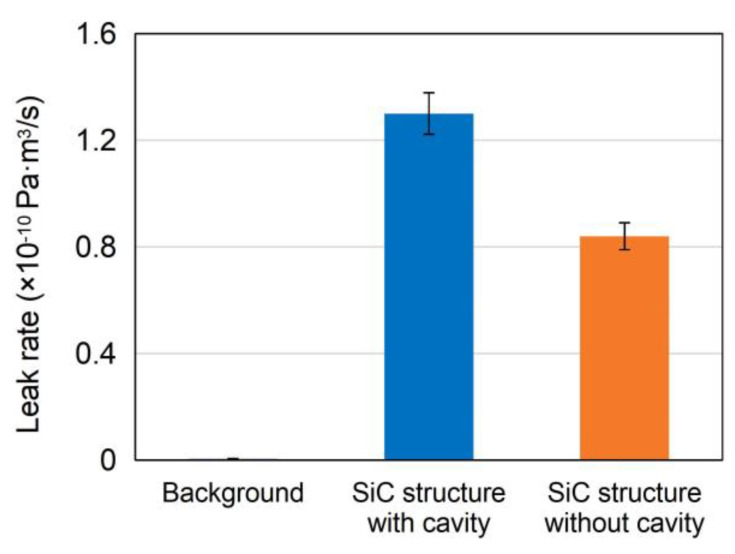
Measured leak rates of fine leak detection.

The quantitative analysis of leak rate of the cavity structure is performed through leakage detection, which is of great significance to the long-term stability for high temperature applications. First, the fine leak detection consists of a helium pressurization stage followed by helium leak detection with a mass spectrometer. The sample was kept in a pressure chamber filled with helium at a pressure of 517 kPa for an exposure time of 2 h. After that, the sample was removed from the chamber and transferred to the helium mass spectrometer (AHNOY NHJ-600) after a certain dwell time. As shown in Figure 9a, the results show that the measured leak rate of the sample is 1.3 × 10^−10^ Pa⋅m^3^/s, which is less than the failure criteria of 5 × 10^−9^ Pa⋅m^3^/s. This means that the SiC cavity structure can meet the requirement of fine leak detection in the specification. Based on Formula (10), the equivalent standard leak rate of cavity structure is 1.1 × 10^−10^ Pa⋅m^3^ s^−1^. To further eliminate the measurement error caused by large leakage, the sample with fine leak detection was again tested by the coarse leak detection. The sample was placed in a vacuum chamber for 30 min. Then the sample was immersed in light fluorine oil and pressurized with nitrogen at a pressure of 617 kPa for 1 h. Subsequently, the sample was removed from the chamber and dried in air for a certain time prior to immersion in a fluorine oil heater (ARMSTECH A603) containing heavy fluorine oil of 125 °C. As shown in Figure 9b, no bubble could be observed under the illumination of the lighting source, which indicates that there is no large leakage in the sealed cavity structure. Moreover, the leak rate of SiC structure without cavity was also measured and the measured leak rate was 8.5 × 10^−11^ Pa m^3^ s^−1^, as shown in Figure 10. It is inferred that there might still be some inconspicuous voids in the amorphous layer of the bonding interface due to the absence of subsequent rapid thermal annealing. These voids become temporary storage areas of helium, resulting in the leakage of the SiC structure without cavity. In the actual manufacturing process of SiC pressure sensor, high temperature rapid thermal annealing in inert gas is widely used in the ohmic contact formation, which could cause the intermediate amorphous layer of the bonding interface to recrystallize. It is beneficial to decrease the leak rate and further improve the airtightness of cavity structure.

## 4. Conclusions

In this paper, a SiC cavity structure is achieved based on room temperature surface activated bonding (SAB) as the basis for an all-SiC piezoresistive pressure sensor. For bonding demonstration, a bare SiC wafer is bonded to another patterned SiC substrate with etched shallow grooves, which avoids the time-consuming and undesirable deep etching process. Moreover, the room temperature direct bonding technique avoids the energy consumption caused by a high temperature bonding process and the thermal stress caused by the mismatch in thermal expansion coefficients of different materials. Subsequently, the hermeticity analysis on SiC cavity structure was carried out. The microstructure observation indicates that the SiC cavity structure is tightly bonded and remains intact. Furthermore, the average tensile strength of bonding interface could reach ~8.01 MPa through the tensile testing. Moreover, the high quality hermetic sealing of SiC cavity structure has been achieved by the SAB method proved by the leakage detection, which is important for the long-term stability of absolute pressure sensors. Therefore, the SiC cavity structure based on room temperature direct bonding process can be potentially further developed into an all-SiC piezoresistive pressure sensor for high temperature applications. Future work will focus on the manufacturing of all-SiC piezoresistive pressure sensors based on the research results of this paper.

## Data Availability

The data presented in this study are available on request from the corresponding author.
